# circCPA4 acts as a prognostic factor and regulates the proliferation and metastasis of glioma

**DOI:** 10.1111/jcmm.14541

**Published:** 2019-08-19

**Authors:** Hao Peng, Chaoying Qin, Chao Zhang, Jun Su, Qun Xiao, Yao Xiao, Kai Xiao, Qing Liu

**Affiliations:** ^1^ Department of Neurosurgery, Xiangya Hospital Central South University Changsha China

**Keywords:** ceRNA, circRNA, glioma, metastasis, proliferation

## Abstract

Circular RNAs (circRNAs) are reported to play vital roles in tumour process and might be potential prognostic biomarkers and therapeutic targets for tumours. But the expression and function of circRNAs in glioma remain unclear. Here, we performed circRNA microarray analysis of glioma tissues and matched normal brain tissue samples to explore the circRNA profile in glioma. GO analysis, KEGG and Reactom pathway analysis of linear mRNA transcripts corresponding to circRNAs were performed to study the involved biological process and pathways. The clinical significance of the selected circRNA was investigated by Kaplan‐Meier survival analysis. Relevant biological function, such as cell proliferation and metastasis, was detected in vitro and in vivo*.* And possible mechanism of the regulatory function of the selected circRNA in glioma was explored. We found that circCPA4 (hsa_circ_0082374) up‐regulated the most in glioma tissues and high levels of circCPA4 were positively related to poor outcome of glioma. And knockdown of circCPA4 suppresses cell proliferation and metastasis in glioma. Moreover, circCPA4 interacts with let‐7 and serves as a sponge for let‐7. Through the competitive endogenous RNA (ceRNA) mechanism, circCPA4 sponges let‐7 to regulate the expression of CPA4 and glioma progression. The circCPA4/let‐7/CPA4 axis regulates glioma progression by ceRNA mechanism, and circCPA4 could be a novel prognostic biomarker and target for glioma treatment.

## 1 INTRODUCTION

Glioma is the most common malignant primary brain tumour and the deadliest form of brain tumour in adults.^1^, ^2^ Despite the advances in surgery and adjuvant therapies, the prognosis of glioma remains poor. Studying the potential mechanisms of glioma is important for glioma therapy. Novel targets are needed to achieve a better anti‐tumour response.

It has been reported that circular RNAs (circRNAs) are involved in tumour progress.^3^, ^4^ Through their regulatory functions, circRNAs could act as oncogenes or tumour suppressors in tumour progression, which indicates that circRNAs could be novel biomarkers or targets for tumour treatment.^5^, ^6^ However, the expression and function of circRNAs in glioma are still unclear.

Competitive endogenous RNA (ceRNA) is the most studied field of circRNA function. RNAs, circRNAs included, can serve as ceRNAs and compete binding to shared miRNAs.^7^ Due to their stability, predominant cytoplasm localization and the non‐coding nature, circRNAs have advantages as ceRNAs.^8^ There are various studies reporting circRNAs acting as ceRNAs to regulate tumour progress.^9^ In colon cancer, circRNA CCDC66 acts as a ceRNA to sponge miRNAs and promotes tumour growth and metastasis.^10^ Additionally, circRNA circPVT1 sponges several tumour suppressor miRNAs such as let‐7b to suppress senescence.^11^ Considering the important roles circRNAs play in tumours, we are urged to investigate the function of circRNAs as novel biomarkers or targets for glioma treatment.

In this study, we performed circRNA microarray analysis of glioma tissues and matched normal brain tissue samples to explore the circRNA profile in glioma. GO analysis, KEGG and Reactom pathway analysis of linear mRNA transcripts corresponding to circRNAs were performed to study the involved biological process and pathways. The clinical significance, relevant biological function and possible mechanism of the selected circRNA were investigated.

## 2 MATERIAL AND METHODS

### 2.1 Ethical standards

This study was approved by the Ethics Committee of Xiangya Hospital, Central South University, and performed accordingly with the Declaration of Helsinki. Written informed consents are provided by participants. Animal studies were approved and performed accordingly with the guidelines of the Institutional Animal Care and Use Committee (IACUC) of Xiangya Hospital, Central South University.

### 2.2 Patient samples

Glioma tissues and matched normal brain tissue samples were collected from Xiangya Hospital and submitted to circRNA microarray analysis. Glioma tissues of 73 patients were collected from Xiangya Hospital and submitted to qRT‐PCR.

### 2.3 Microarray analysis

Glioma tissues and matched normal brain tissue samples were submitted to circRNA microarray analysis using CapitalBio Technology Human CircRNA Array v2. The circRNA array data were analysed by GeneSpring software V13.0 (Agilent). The result was log2‐transformed and median centred by genes with CLUSTER 3.0 software and analysed with hierarchical clustering by average linkage.

### 2.4 Quantitative real‐time PCR (qRT‐PCR)

RNA was isolated by TRIzol (Invitrogen). Nuclear and cytoplasmic fractions were isolated by NE‐PER Nuclear and Cytoplasmic Extraction Reagents (Thermo Scientific). qRT‐PCR was performed with SYBR Premix Ex Taq (Takara Bio Inc) and All‐in‐One™ miRNA qRT‐PCR Detection Kit (GeneCopoeia). Primers were synthesized by Invitrogen (Table S1).

### 2.5 Cell culture and transfection

Glioma cell lines U251 and U87 were purchased from American Type Culture Collection. Cells were cultured accordingly with the supplier's instructions. Cell authenticity was verified by DNA fingerprinting.

Cells were transfected using Lipofectamine 2000 (Invitrogen). siRNAs targeting circCPA4, let‐7 mimics and inhibitors were purchased from GeneCopoeia (Table S1).

### 2.6 Cell counting kit‐8 (CCK‐8) assay

Cells (1 × 10^3^) were seeded, and CCK‐8 solution (10 μL, Dojindo Laboratories) was added 48 hours after transfection. The absorbance at 450 nmol/L was detected with Bio‐Tek EPOCH2 (USA) after incubation at 37°C for 2 hours.

### 2.7 Colony formation assay

Cells (1 × 10^3^) were seeded and incubated for 2 weeks. Then, the colonies were fixed by methanol for 10 minutes and stained by 0.1% crystal violet for 15 minutes. After that, the colonies were imaged and counted.

### 2.8 Transwell assay

Cells (1 × 10^4^) were seeded in migration chambers (BD Biosciences), and culture medium containing 10% FBS was added to the lower chamber. Invasive cells were fixed by methanol for 10 minutes and stained by 0.1% crystal violet for 15 minutes 24 hours after seeding. After that, the invasive cells were imaged and counted.

### 2.9 Mouse xenograft model

Cells (2 × 10^6^) were subcutaneously injected into 4‐week‐old BALB/c nude mice (three mice per group). The mice were treated with an intratumoural injection (40 μL si‐NC or si‐circCPA4) every 4 days. After 28 days, the xenograft tumours were excised and tumour weights were detected.

For lung metastasis, cells (1 × 10^5^) were injected through tail veins (five mice per group). Then, the lungs were excised and the numbers of metastatic nodules were counted after 8 weeks.

### 2.10 Luciferase reporter assay

Cells (5 × 10^3^) were transfected with corresponding plasmids and let‐7 mimics using Lipofectamine 2000. And luciferase activities were quantified with a dual‐luciferase reporter assay system (Promega) 48 hours after transfection.

### 2.11 RNA immunoprecipitation (RIP) assay

Cells were transfected with MS2bs‐circCPA4, MS2bs‐circCPA4mt or blank control MS2bs‐Rluc using Lipofectamine 2000. RIP was performed by a GFP antibody (Roche) and a Magna RIP RNA‐Binding Protein Immunoprecipitation Kit (Millipore) 48 hours later. For RIP assay on Ago2, RIP was performed by an anti‐Ago2 antibody (Millipore) 48 hours after transfection, and the levels of circCPA4, CPA4 and let‐7 were measured.

### 2.12 Western blotting

Briefly, proteins were separated by 10% SDS‐PAGE and then transferred to PVDF membranes (Millipore). The membranes were incubated with 5% skim milk and then incubated with antibody against CPA4 (1:1000, Abcam), followed by incubation with an HRP‐labelled secondary antibody (CST) and detection by chemiluminescence. An anti‐β‐actin antibody (1:1000, Affinity, USA) served as control.

### 2.13 Statistical analysis

Statistical analysis was performed using SPSS 19.0 software. Comparisons between groups were analysed using *t* tests. Survival analysis was performed with Kaplan‐Meier plots and log‐rank tests. Data are presented as mean ± SD of three independent experiments. *P* < .05 means statistically significant.

## 3 RESULTS

### 3.1 circCPA4 is up‐regulated and correlated with poor outcome of glioma

**Figure 1:**
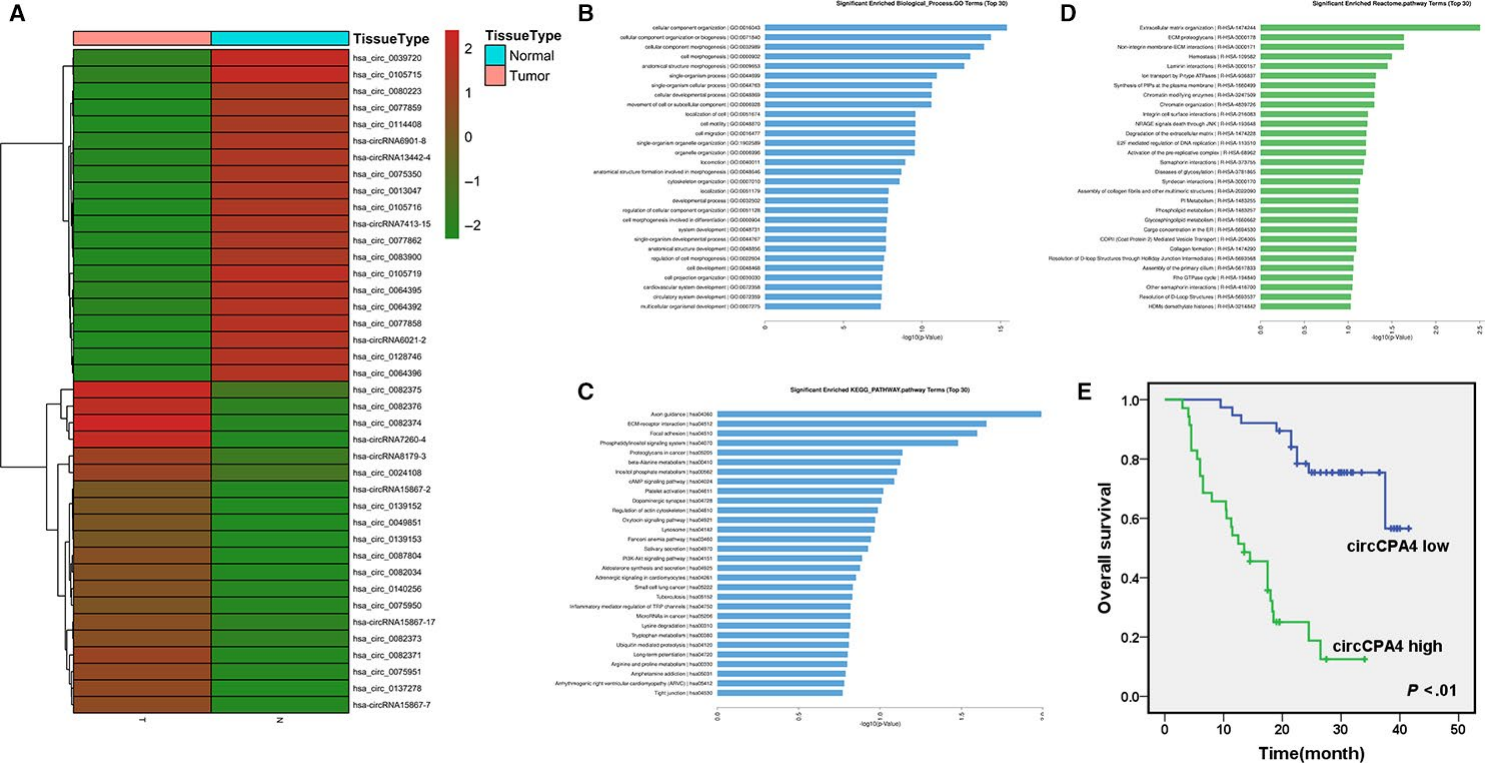
circCPA4 is up‐regulated and correlated with poor outcome of glioma A, Hierarchical cluster analysis showed the top 20 up‐regulated and down‐regulated circRNAs in glioma tissues and matched normal brain tissue samples: red, up‐regulated; green, down‐regulated. B, GO analysis was performed. C, KEGG pathway analysis was performed. D, Reactome pathway analysis was performed. E, OS curves for 73 glioma patients with high or low circCPA4 expression

To explore the potential involvement of circRNAs in glioma, high‐throughput circRNA microarray assays of glioma tissues and matched normal brain tissue samples were performed. In glioma tissues, 14754 circRNAs were down‐regulated and 13511 circRNAs were up‐regulated at least 2‐fold. Figure 1A showed the top 20 up‐regulated and down‐regulated circRNAs.

GO analysis, KEGG and Reactom pathway analysis of linear mRNA transcripts corresponding to circRNAs were conducted. GO analysis revealed that the corresponding linear mRNAs were related to cell motility and cell migration (Figure 1B). KEGG pathway analysis indicated focal adhesion and tight junction (Figure 1C). Reactom pathway analysis also indicated collagen formation and degradation of extracellular matrix (Figure 1D). Among the top 20 up‐regulated circRNAs, hsa_circ_0082374 up‐regulated the most in glioma tissues and we decided to study this circRNA. Hsa_circ_0082374 (chr7: 129948146‐129964020) was assumed to derive from carboxypeptidaseA4 (CPA4) by human reference genome (GRCh37/hg19). Thus, we named hsa_circ_0082374 as ‘circCPA4’.

To explore the clinical significance of circCPA4 in glioma, we performed survival analysis on 73 glioma patients. CircCPA4 expression equalled to or greater than the average was considered as ‘circCPA4 high’ group (35/73). Kaplan‐Meier survival analysis revealed that high levels of circCPA4 were related to poor outcome of glioma (Figure 1E).

### 3.2 Inhibition of circCPA4 suppresses glioma proliferation and metastasis

**Figure 2:**
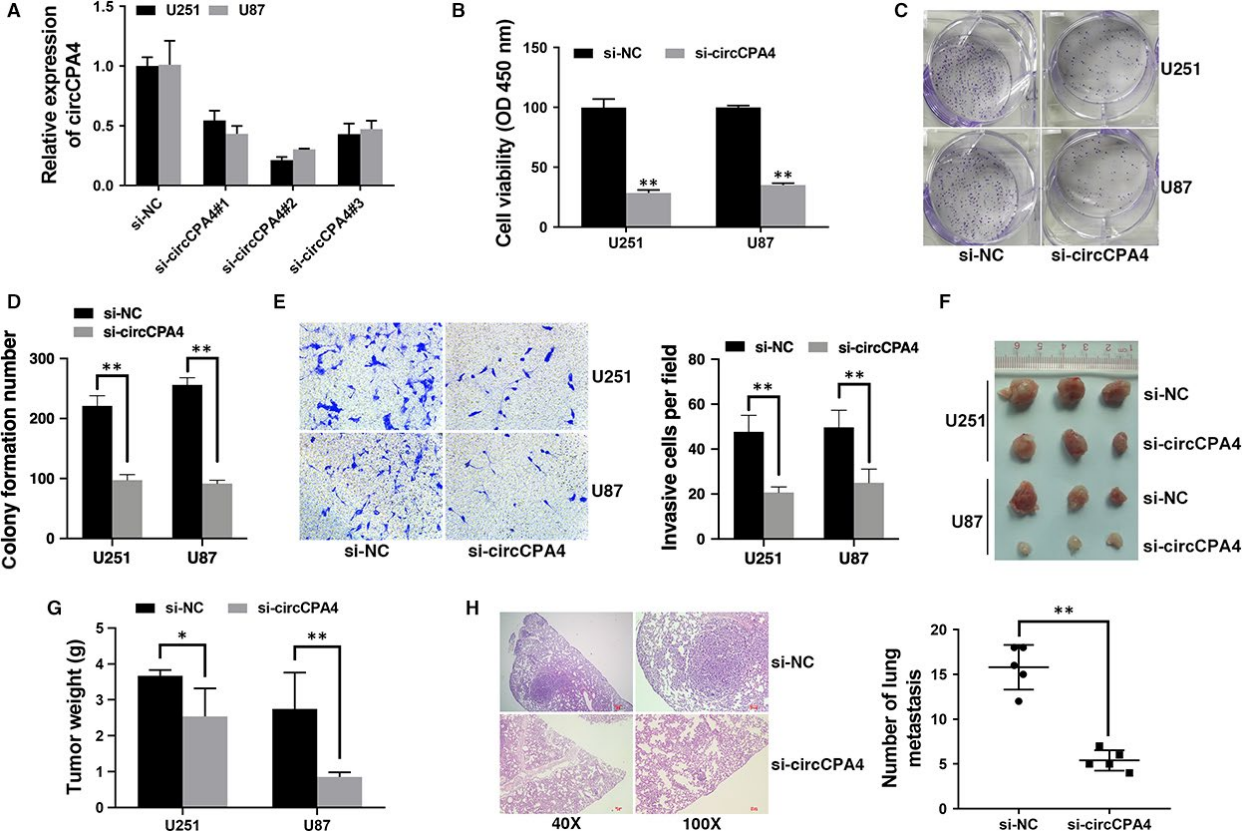
Inhibition of circCPA4 suppresses glioma proliferation and metastasis A, si‐circCPA4#2 successfully knocked down circCPA4. B, CCK‐8 assay was performed to assess cell proliferation. C, Colony formation assay was performed to assess cell colony‐forming ability. D, Colony formation number was quantified by ImageJ. E, Transwell assay was performed to assess cell invasive ability (left). The number of invasive cells was quantified by ImageJ (right). Original magnification, x100. F, Xenograft models were established. G, Summary of tumour weights. H, Representative images of HE‐stained lung metastatic nodules (left). The number of metastatic nodules was quantified (right). *P < .05, **P < .01

To investigate the function of circCPA4 in glioma, circCPA4 was knocked down by si‐circCPA4#2 (Figure 2A). Inhibition of circCPA4 suppressed cell proliferation (Figure 2B) and reduced cell colony formation ability (Figure 2C,D). And invasive cells were reduced after inhibition of circCPA4 (Figure 2E). To investigate the function of circCPA4 in vivo, we established mouse xenograft models. Inhibition of circCPA4 suppressed tumour growth (Figure 2F,G) and lung metastasis (Figure 2H), indicating that inhibition of circCPA4 suppresses proliferation and metastasis in glioma.

### 3.3 circCPA4 acts as a sponge for let‐7

**Figure 3:**
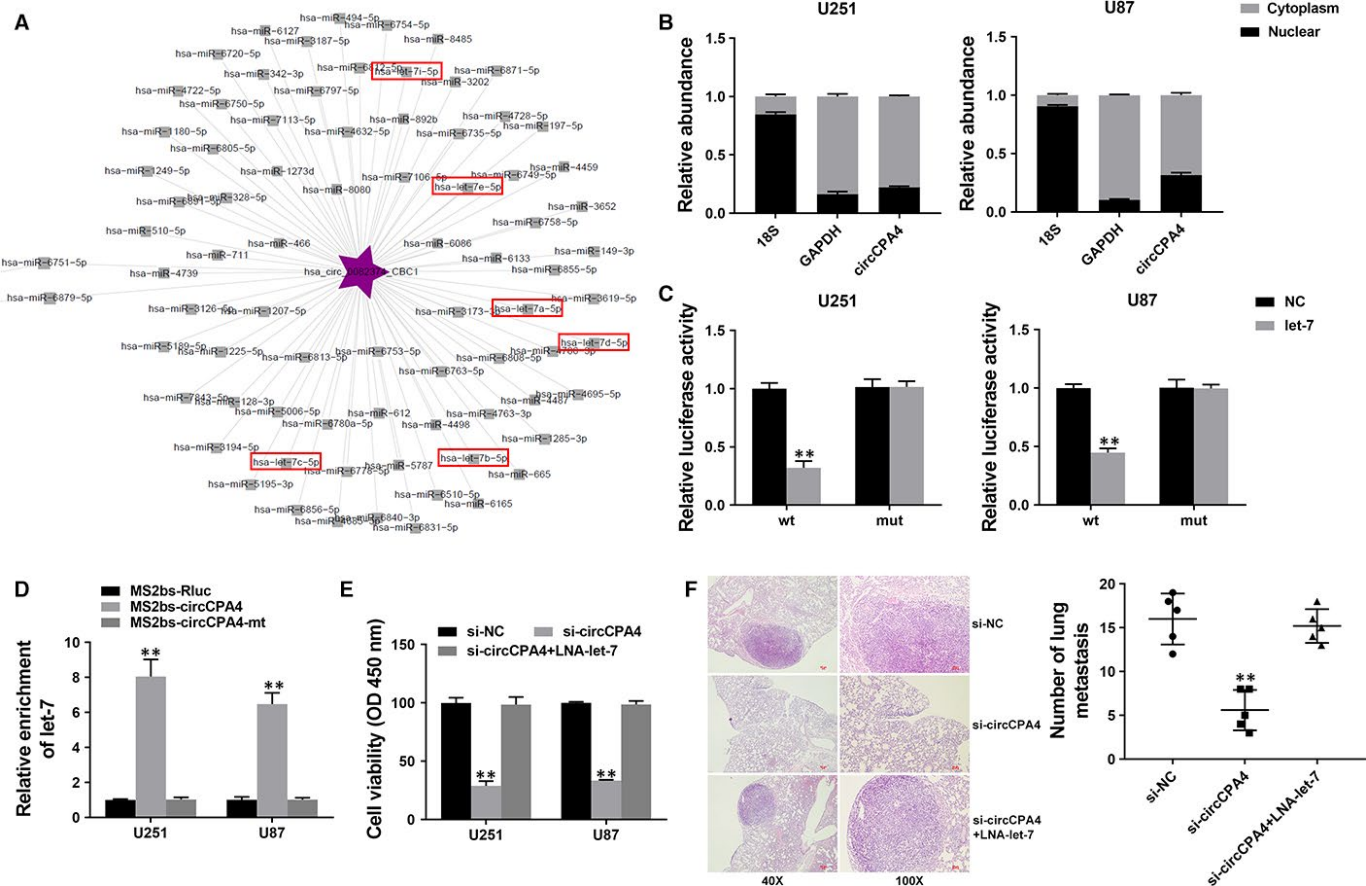
circCPA4 acts as a sponge for let‐7 A, The predicted target miRNAs of circCPA4. B, The levels of nuclear control transcript (18S), cytoplasmic control transcript (GAPDH) and circCPA4 were assessed in nuclear and cytoplasmic fractions. C, Luciferase assay of cells cotransfected with let‐7 mimics and wild‐type or mutant luciferase reporter. D, MS2‐based RIP assay in cells transfected with MS2bs‐circCPA4, MS2bs‐circCPA4mt or control. E, CCK‐8 assay was performed to assess cell proliferation. F, Representative images of HE‐stained lung metastatic nodules (left). The number of metastatic nodules was quantified (right). **P < .01

CircRNAs are involved in transcriptional controlling by serving as ceRNAs. The circRNA‐miRNA network analysis showed that circCPA4 has binding sites for let‐7 (Figure 3A). Thus, we explored the intracellular location of circCPA4. We found it predominantly localized in the cytoplasm, indicating that circCPA4 might serve as sponge for miRNAs (Figure 3B). Thus, we performed luciferase reporter assay and found that the luciferase activity decreased after co‐transfection of wild‐type luciferase reporter and let‐7 mimics (Figure 3C). To further validate the binding of circCPA4 and let‐7, we continued to perform RIP assay and found that let‐7 was mostly enriched in MS2bs‐circCPA4 group (Figure 3D), indicating that circCPA4 could directly interact with let‐7 and serve as a sponge for let‐7.

To confirm that circCPA4 acts by sponging let‐7, we continued to perform rescue experiments. CCK‐8 assays showed that the suppressed cell proliferation induced by inhibition of circCPA4 was reversed by let‐7 inhibitor (Figure 3E). And reduced lung metastasis induced by circCPA4 knockdowned was reversed by let‐7 inhibitor (Figure 3F), suggesting that circCPA4 acts by sponging let‐7 in glioma.

### 3.4 circCPA4 acts as a ceRNA to regulate CPA4

**Figure 4:**
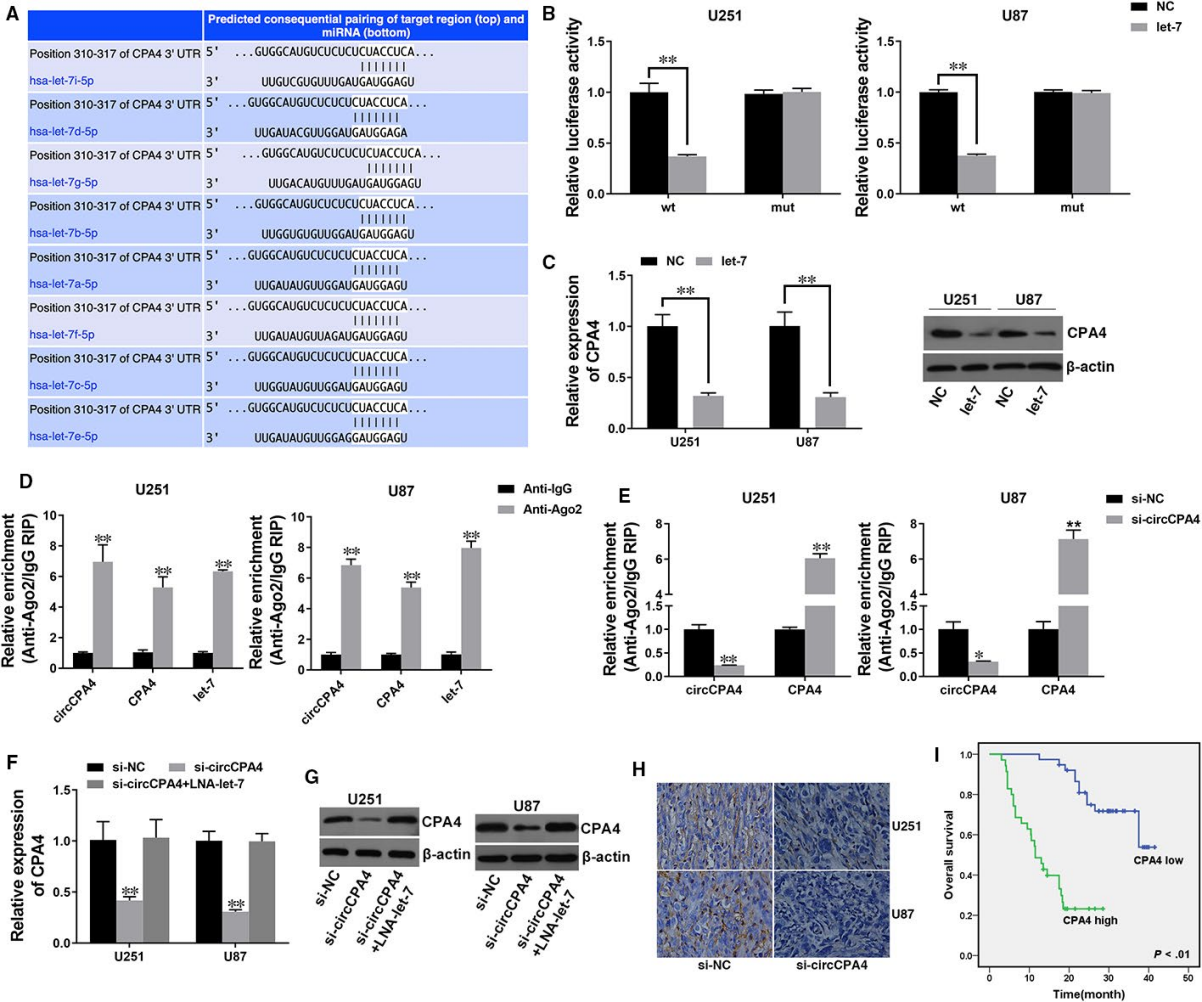
circCPA4 acts as a ceRNA to regulate CPA4 A, The predicted binding sites of let‐7 within the CPA4 3'UTR. B, Cells were transfected as described, and luciferase assay was performed. C, Cells were transfected as described, and the expression of CPA4 was determined by Western blotting (left) and quantified by ImageJ (right). D, Cells were transfected as described, and the expression of CPA4 was determined by qRT‐PCR. E, RIP assay showing the enrichment of circCPA4, CPA4 and let‐7 on Ago2 relative to IgG. F, Cells were transfected as described, and a RIP assay on Ago2 was performed. G, Cells were transfected as described, and the expression of CPA4 was determined by qRT‐PCR. H, The expression of CPA4 was determined by Western blotting (left) and quantified by ImageJ (right). I, The expression of CPA4 in xenograft tumours was determined by immunohistochemistry. J OS curves for 73 glioma patients with high or low CPA4 expression. **P < .01

To explore whether circCPA4 sponges let‐7 to liberate its target's expression, we used TargetScan to find target genes of let‐7 and found CPA4 (Figure 4A). We performed luciferase reporter assay and found reduced luciferase activity after co‐transfection of let‐7 mimics and wild‐type luciferase reporter (Figure 4B). Additionally, let‐7 could suppress the expression of CPA4, indicating that CPA4 is a downstream target of let‐7 and could be regulated by let‐7 (Figure 4C,D).

RNA immunoprecipitation assay on Ago2 revealed that circCPA4, CPA4 and let‐7 were mostly enriched to Ago2, suggesting that circCPA4 and CPA4 are recruited to Ago2‐related RISC and interact with let‐7 (Figure 4E). Additionally, knockdown of circCPA4 reduced Ago2 enrichment to circCPA4, but increased Ago2 enrichment to CPA4, suggesting that circCPA4 acts as a ceRNA and compete binding to miRNAs with CPA4 (Figure 4F). Moreover, inhibition of circCPA4 decreased the expression of CPA4, which could be reversed by let‐7 inhibitor, indicating that circCPA4 sponges let‐7 to regulate the expression of CPA4 (Figure 4G,I). Next, we explored the clinical significance of CPA4 in glioma and found that high CPA4 expression (35/73) was related to poor outcome of glioma (Figure 4J).

## 4 DISCUSSION

Recent studies suggest that circRNAs play vital roles in tumour procession, including cell apoptosis, angiogenesis and cell cycle progression.^12^, ^13^ And circRNAs could be promising prognostic biomarkers and therapeutic targets in tumour treatment.^14^, ^15^ But the expression and function of circRNAs in glioma are still not clear. A study revealed that more than 476 circRNAs were differentially expressed in glioma.^16^ And circ_002136 was found to inhibit the viability, migration and tube formation of glioma.^17^ Here, we performed high‐throughput circRNA microarray assays of glioma tissues and matched normal brain tissue samples to investigate the involvement of circRNAs in glioma. The result showed that circCPA4 (hsa_circ_0082374) up‐regulated the most in glioma and high levels of circCPA4 were positively associated with poorer survival of glioma (Figure 1). Moreover, circCPA4 was involved in glioma progression, and inhibition of circCPA4 suppressed glioma proliferation and metastasis (Figure 2). These results suggested that circCPA4 could be a novel prognostic biomarker and target for glioma treatment.

It has been reported that circRNA could act as ceRNA to regulate tumour progression.^18^ CircRNA circHIPK3 was reported to regulate cell growth via sponging multiple miRNAs.^19^ In hepatocellular carcinoma, circRNA circMTO1 and cSMARCA5 were found to serve as miRNA sponges and regulate tumour progression.^20^, ^21^ However, no such study has been reported in glioma. Here, we used the circRNA‐miRNA network analysis to explore the target miRNAs of circCPA4 and found binding sites for let‐7. Let‐7 has long been reported to function as tumour suppressor and is involved in multiple tumour processes.^22^ Let‐7 could influence genomic stability and is related to survival after anticancer cytotoxic therapy.^23^, ^24^ However, its involvement in glioma remains mostly unclear. It has been reported that let‐7 suppressed the expression of MDM4 after DNA damage in glioma.^25^ Here we found that circCPA4 could directly interact with let‐7 and function through sponging let‐7 (Figure 3).

Then, we used TargetScan to explore potential target genes of let‐7 and found CPA4. The aberrant expression of CPA4 is associated with cancer progression. It has been reported to involve in prostate cancer aggressiveness.^26^ In pancreatic cancer, CPA4 was significantly overexpressed and closely related to tumour progression and poor prognosis.^27^ In gastric cancer, CPA4 was highly expressed and could be an independent poor prognostic factor.^28^ In non–small‐cell lung cancer, high CPA4 expression is related to poor outcome and could be used in early detection.^29^ In breast cancer, CPA4 was reported to be a prognosis marker and therapeutic target.^30^ However, CPA4 expression and clinical significance in glioma have not yet been reported. Here, we found that CPA4 is a downstream target of let‐7 and could be regulated by let‐7. And circCPA4 acted as a ceRNA to compete binding to let‐7 with CPA4 to regulate the expression of CPA4. Survival analysis revealed that high levels of CPA4 were related to poor outcome of glioma (Figure 4).

In short, circCPA4 is up‐regulated in glioma and related to poor survival of glioma. CircCPA4 could regulate the proliferation and metastasis of glioma, and regulate CPA4 expression through sponging let‐7 in glioma. The circCPA4/let‐7/CPA4 axis regulates glioma progression by ceRNA mechanism, and circCPA4 could be a novel biomarker and target for glioma treatment.

## CONFLICTS OF INTEREST

The authors declare no conflict of interest.

## AUTHORS' CONTRIBUTIONS

QL designed the experiments. HP, CQ and CZ performed the experiments. JS and QX analysed and interpreted the data. YX and KX were the major contributors in writing the manuscript. All authors read and approved the final manuscript.

## Data Availability

The data that support the findings of this study are available from the corresponding author upon reasonable request.
